# Has the COVID-19 pandemic lockdown worsened eating disorders symptoms among patients with eating disorders? A systematic review

**DOI:** 10.1007/s10389-022-01704-4

**Published:** 2022-03-29

**Authors:** Yunqi Gao, Nasser Bagheri, Luis Furuya-Kanamori

**Affiliations:** 1 Centre for Research on Ageing, Health & Wellbeing, Australian National University, Acton, ACT 2601 Australia; 2 Centre for Mental Health Research, Australian National University, Acton, ACT 2601 Australia; 3 The Visual and Decision Analytics (VIDEA) Lab, Health Research Institute, The University of Canberra, Bruce, Australia; 4 UQ Centre for Clinical Research, The University of Queensland, Herston, QLD 4029 Australia

**Keywords:** coronavirus, lockdown, eating disorders, anorexia nervosa, bulimia nervosa, binge eating disorder

## Abstract

**Objective:**

During the coronavirus pandemic lockdowns, general medical complications have received the most attention, and few studies have examined the association between the COVID-19 lockdown and eating disorders (ED). This study aimed to investigate the impact of the coronavirus lockdowns on ED symptoms severity and summarize factors associated with lockdowns that led to changes in eating disorders.

**Method:**

PubMed, Scopus, and Cochrane Library databases were searched for studies measuring the impact of coronavirus lockdowns on ED symptoms.

**Results:**

A total of 132 studies were retrieved, after abstract screening and removal of duplicates, 21 papers were full-text screened, and 11 eligible papers were identified. Factors associated with symptomatic deterioration in ED patients during COVID-19 lockdowns included disruption of lifestyle routine, social isolation, reduced access to usual support networks, limited or no access to healthcare and mental care services, and social anxiety.

**Discussion:**

Overall, the pandemic lockdowns were associated with worsening of eating disorders.

This triggering environment can lead to increased anxiety and depression symptoms, change in dietary habits, and eventually result in worsening eating disorder symptoms.

## Introduction

The novel coronavirus disease 2019 (COVID-19) was first reported in Wuhan, China (Temsah et al. 2020). The World Health Organization (WHO) declared that the outbreak was considered a global pandemic on 11 March 2020 (Anand et al. 2020). The rapid increase of confirmed cases and the death rate led to implementing public health measures to prevent transmission. Some important measures included lockdown, travel restrictions, and physical distancing. Apart from the growing financial loss in industries (Nicola et al. 2020), the economic downturn resulted in an increased unemployment rate and had an adverse impact on the quality of life (Roca et al. 2013). Unlike other infectious diseases, the pandemic influenced both physical and mental wellbeing (Hasan and Bao 2020). Fear of uncertain prognoses, shortage of testing and treatment resources, unexpected lockdowns, and social isolation may precipitate psychological distress and mental illness across the population (Mamun and Ullah 2020; Wang et al. 2020a, b), especially in people with pre-existing mental health difficulties (Holmes et al. 2020). With increased feelings of isolation and loneliness during the coronavirus pandemic and lockdown, patients with eating disorders may face an even greater challenge for recovery. A study by Touyz et al. (2020) concluded that eating disorders involve a problematic and unhealthy relationship with food, and the symptoms may be enhanced during the disease outbreak due to food insecurity and panic buying.

Countries and territories worldwide have put public health measure in place (e.g., lockdown, mandatory face coverings) to decrease the spread of the coronavirus across communities (Graell et al. 2020; Orgilés et al. 2020). The COVID-19 lockdowns might trigger eating disorder symptomatology in four aspects. First is the disruption of the living situation. Travel restrictions, limited access to healthcare services, and grocery stores might cause sudden lifestyle changes and social isolation in people suffering from eating disorders. Naja & Hamadeh’s (2020) study concluded that the lockdown measures might induce irregular eating habits and sedentary behaviors. Several countries have issued the recommendation to limit grocery trips during the pandemic to promote social distancing in public places (Ruth et al. 2020), and this might encourage patients with eating disorders to purchase excess quantities of food. Eating disorder patients experienced limited access to their usual support networks during the enforced lockdown, which led to a reduced motivation for recovery and worsened eating disorder symptoms. Second is the increasing consumption of social media. Technology is vital during this pivotal time, as physical associations and outdoor activities are limited and people tend to rely on the internet. However, media can influence disordered eating in many ways. Eating disorder levels might worsen through exposure to unrealistic body image, negative stories, and stressful news (Rodgers et al. 2020). Anxiety during quarantine and weight stigma can contribute to raising body shame and disordered eating (Robertson et al. 2021), particularly in those with a current or historic eating disorder. In addition, accessibility to food can exacerbate irregular eating behaviors. According to a UK household food security survey, 16.2% of participants have experienced food insecurity since the lockdown, while 21.6% worried about the availability of food (Loopstra 2020). Population with a higher level of food insecurity are more likely to endorse binge eating and obesity (Becker et al. 2017; Rasmusson et al. 2018). A food frequency questionnaire in Denmark discovered that people tended to change their food-related habits during the pandemic, and showed an increasing preference for food high in sugar and fat (Janssen 2020). In general, the average sales of frozen food, cakes, snacks, and other shelf-stable foods have increased since the start of the pandemic, while the consumption of fresh fruit and vegetables has decreased. Lastly is the negative emotions related to the coronavirus and the unexpected lockdown, which might lead to an increasing level of stress and changes in appetite for patients with eating disorders (ED) (Rodgers et al. 2020). Therefore, it is critical to analyze the impact of the COVID-19 pandemic-related lockdowns on disordered eating behaviors, and there is an urgent need for interventions to support these vulnerable populations.

The impact of lockdown measures is interrelated with the coronavirus pandemic, and this research work focused on the association between the COVID-19 pandemic-related lockdown and eating disorders. This study aimed to systematically review the evidence on eating disorders and the coronavirus lockdowns, investigate factors impacting changes in ED levels due to the COVID-19 lockdowns, and generate an overview of the association between ED symptom severity and the global pandemic. The specific objectives of the study were:

1. To examine if eating disorder symptoms worsened during the COVID-19 lockdown?
2. To describe what factors of the coronavirus lockdowns impacted eating disorder symptoms (e.g., social isolation, food insecurity)?

## Methodology

### Literature search

The literature review was carried out in the following databases: PubMed, Scopus, and Cochrane Library. Reference lists of relevant articles were also searched. The following search terms were used: “coronavirus” OR “COVID-19” OR “SARS-CoV-2” AND “lockdown” AND “eating disorders”. Searches were conducted up to April 2021, all relevant articles published between the start of the pandemic in 2019 through April 2021 were considered for inclusion.

### Eligibility criteria

The inclusion criteria incldued epidemiological studies on patients with diagnosed eating disorders and reported changes in ED symptoms severity (either self-reported or through medical records) before and after COVID-19 lockdown. Exclusion criteria included study populations without a primary diagnosis of eating disorders or did not contain a comparison of ED symptoms before and during the lockdown. For studies that included both healthy individuals and ED patients, we only focused on the outcome for the ED group. Review articles, editorials, and opinions were excluded. No restrictions on the region of publication, publishing language, gender, or the age of the patients were imposed.

### Study selection and data collection procedure

First duplicate articles were excluded, then titles and abstracts, followed by full-text article screening were applied to identify articles that met our inclusion criteria. Data were extracted by the main author and search outcomes were imported to Endnote for review. The following data were extracted from the studies: author name, year of publication, demographic and methodological characteristics (e.g., mean age and gender of the study participants, sample size, study design), diagnosis (anorexia nervosa, bulimia nervosa, binge eating disorder, and other specified eating disorder), and description of the outcomes.

### Quality assessment

The main author did the quality assessment of all studies (n=11) using the Newcastle-Ottawa Scale (Wells et al. 2000). There are three domains considered in the scale: selection, comparability, and outcome. All 11 studies satisfy the minimum ratings for the review, with 4 having a fair quality rating, and 7 a good quality rating.

## Results

### Study selection

The systematic search identified 132 articles presenting outcomes for individuals with eating disorder symptoms during the COVID-19 pandemic lockdowns. After the removal of duplicates, 107 studies remained. Eighty-six studies did not meet the inclusion criteria and were discarded after reviewing the title and abstract. The remaining 21 studies were full-text screened, and 11 studies were considered eligible for the systematic review. Two studies, Graell et al. (2020) and Castellini et al. (2020) included both individuals with and without EDs, hence only data on ED patients were extracted from these studies.

**Fig. 1:**
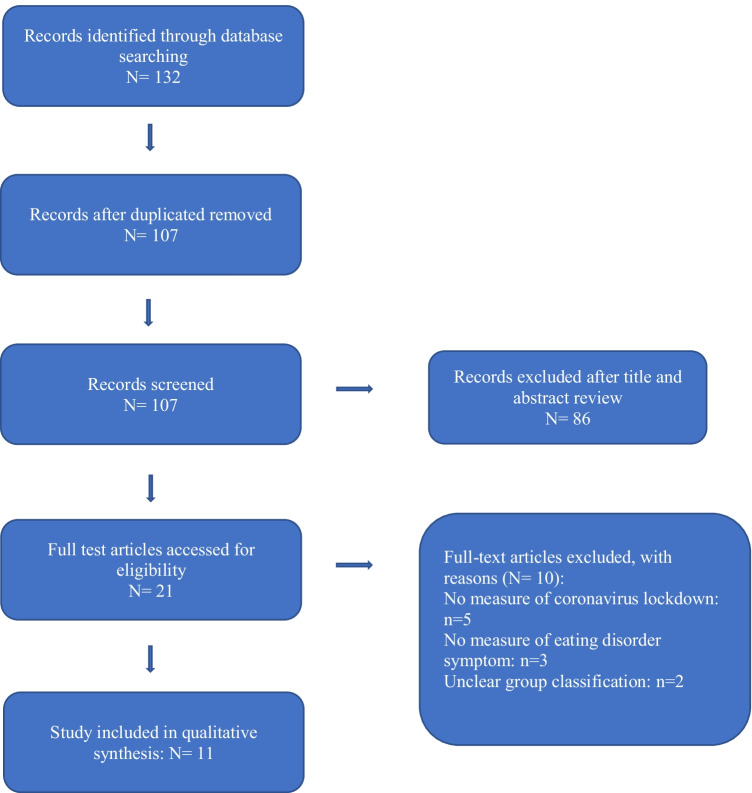
Flow chart for study inclusion and exclusion

Eleven papers report the association between the COVID-lockdown measures and ED symptoms, participants’ information was collected online or via telephone surveys, video conferences, and face-to-face interviews. Nine out of 11 studies were cohort studies and compared participants’ ED symptoms before and during the COVID-19 lockdown. The survey includes questions on levels of quarantine and changes in eating behavior during the lockdown. Two cross-sectional studies examined participants’ ED patterns during the coronavirus confinement period. Figure 1 presents a flow chart for study inclusion and exclusion, and the details of studies included are summarized in Table 1.

**Table 1:** The impact of lockdown measures on disordered eating symptoms

Authors, Year	Eating disorder type	Location	Sample size	Gender	Mean Age (SD)	Study design	Outcomes
Trott et al. 2021	Eating disorder	England	319	84% female	36.77 (11.75)	Cohort study	Eating disorder symptomology scores have increased significantly post-COVID-19 lockdown.
Graell et al. 2020	Eating disorder	Spain	365 (27 day hospital, 338 outpatient clinic)	87.9% female	Day hospital: 13.18 (3.03) Outpatient clinic: 14.74 (2.33)	Retrospective cohort study, by telephone/ video/ face-to-face	41.9% of participants reported a reactivation of ED symptoms. The majority of clinical worsening is caused by food restriction and social isolation.
Baenas et al. 2020	AN, BN, BED, and other specified feeding or eating disorder	Spain	74	95.9% female	32.12 (12.84)	Case-control study	Around 25% patient experienced worsening ED symptoms during the COVID-19 lockdown. Symptom deterioration is greater among AN patients.
Robertson et al. 2021	Eating disorder	England	264	78% female	Age ranged from 18-79 years, 58% aged 30+ years	Longitudinal cross-sectional study, online survey	35 participants reported a current or part diagnosis of ED. People with pre-existing eating disorders are facing more difficulty in controlling/ regulating eating due to measures associated with lockdown.
Branley-Bell and Talbot 2020	Eating disorder	England	129	93.8% female	29.27 (8.99)	Cross-sectional study, online survey	86.7% report that their eating disorder symptoms have worsened during the pandemic lockdown. 30% of participants believe that their symptoms are much worse.
Leenaerts et al. 2021	BN	Belgium	15	100% female	23	Cohort study	Lockdown measures can worsen the binge eating frequency, especially for patients with more extreme changes in surroundings and social context.
Machado et al. 2020	AN, BN, BED, and other specified feeding or eating disorder	Portugal	43	95.3% female	27.60 (8.45)	Cohort study	31% of participants have gained weight due to COVID-19 lockdown, patients that experienced significant changes in daily routines, tended to develop more disordered eating symptoms and clinical impairment.
Cecchetto et al. 2021	BED	Italy	365	73.1% female	35.09 (13.59)	Cohort study	Lockdown measures have a negative impact on binge eating disorders, loosening restrictions is associated with decreased BED symptoms.
Castellini et al. 2020	AN, BN, eating disorder	Italy	171 (74 patients, 97 health controls)	Not specified	Patients: 31.74 (12.76) Health Control: 30.45 (10.89)	Cohort study	Patients with BN experienced increased symptoms after the lockdown measure. Interestingly, AN patients showed recovery of symptoms during the lockdown.
Monteleone et al. 2021a, b	AN, BN, BED, and other specified feeding or eating disorder	Italy	312	96.2% female	29.19 (12.05)	Retrospective cohort study, online survey	Eating disorder symptoms for participants worsen during the lockdown period, but tend to recover in the following re-opening period.
Herle et al. 2021	Eating disorder	England	22374	76% female	Classified into age groups, majority aged from 46-59 years.	Cohort study	ED patients described lockdown as detrimental, 36% of participants experienced changes in eating behaviors, 3867 reported that they were persistently eating more during the lockdown.

### Impact of COVID-19 lockdown on eating disorders

Most papers (n=7, 63%) adapted screening measures and questionnaires to determine eating disorder symptomology. Trott et al. (2021) used the Eating Attitudes Test 26 (EAT-26) to examine the change in ED during the lockdown, and the cut-off score is greater or equal to 20 (Garner 1991). Trott et al. (2021) compared the eating and exercise behavior pre-COVID-19 and post the first lockdown. The average score for the eating attitude test is significantly higher post-COVID-19, which suggests lockdown and quarantines might trigger higher levels of morbid eating behaviors. Cecchetto et al. (2021) measured eating disorder psychopathology by the 7-Item Binge-Eating Disorder Screener (Herman et al. 2016) and applied the Dutch Eating Behaviour Questionnaire to investigate emotional eating during the pandemic lockdown (Dakanalis et al. 2013). Machado et al. (2020) applied the Eating Disorder Examination-Questionnaire to measure ED symptoms and included specific questions for degrees of lockdown. Similarly, Robertson et al. (2021) requested that participants self-report their mental disorder history. The changes in eating behavior and attitudes during the lockdown were assessed by 5-point Likert Scale survey questions. Branley-Bell and Talbot (2020) adapted the Rumination Response Scale in an online survey, containing both closed and open-ended questions on eating disorders and the COVID-19 pandemic.

Some studies (n=4, 36%) gathered participants’ data by reviewing their medical records. ED patients were diagnosed according to the Diagnostic and Statistical Manual of Mental Disorders, 5th edition (DSM-5) (Baenas et al. 2020; Monteleone et al. 2021a, b; Leenaerts et al. 2021). Monteleone et al. (2021a, b) classified the study into three-time points, which included before coronavirus restrictions, during the pandemic restrictions, and transition from lockdown to reopening. Survey questions related to eating disorders were adapted from the Eating Disorders Inventory (Garner 1991). Graell et al. (2020) and compared patients in day hospitals with those receiving treatment in an outpatient clinic to investigate the impact of therapy cancellation on eating disorders. Anorexia nervosa and restrictive food intake disorder were the more common diseases among adolescent and school-age patients, 41.9% of participants experienced reactivation of ED symptoms during the lockdown period (Graell et al. 2020).

Several of the studies suggested that confinement was closely related to increased eating disorders and anxiety symptoms among the general population (Graell et al. 2020; Termorshuizen et al. 2020; Fernández-Aranda et al. 2020a, b; Herle et al. 2021; Loth et al. 2008). Leenaerts et al. (2021) concluded that participants spent more time at home with their families or housemates due to the nature of enforced lockdowns. The lockdown and quarantine measures led to changes in daily routine, surroundings, and social context, especially for patients with eating disorders. Patients who experienced extreme changes were more vulnerable to negative effects, including increased binge eating frequency (Cecchetto et al. 2021; Leenaerts et al. 2021). The outcome was supported by Machado et al. (2020), the vast majority of participants considered that their daily life were moderately to extremely impacted by the lockdown, which led to emotion regulation problems, limited or no access to healthcare services, and worsened eating disorder symptoms.

Interestingly, several studies reported symptom relief for some participants. In Branley-Bell and Talbot’s (2020) study, the overwhelming majority (86.7%) reported increasing ED symptoms, but two patients reported their symptoms had slightly improved during the lockdown. Baenas et al. (2020) determined that 25.7% of patients reported symptoms worsened during the lockdown, while 51.4% reported their symptoms became less dominant. Patients with AN showed a progressive weight gain and improvement of ED symptoms during lockdown (Castellini et al. 2020). In line with this, a study by Graell et al. (2020) found that approximately 80% of child and adolescent participants report their family relationship has improved during the 8-week confinement period, which is associated with alleviating anorexia nervosa symptoms.

## Discussion

### Summary of the results

Eleven papers examined changes in ED symptom pre vs post-COVID-19 lockdowns, and one study included a following re-opening period. Women and young people had greater concern about their body image and appearance (Williamson et al. 2004; Robertson et al. 2021), faced more difficulties in regulating eating (Robertson et al. 2021), and had a greater risk of worsening eating disorder symptoms during the COVID-19 lockdown (Pierce et al. 2020). In general, all studies concluded that the pandemic lockdown was associated with a worsening of eating disorders, and it led to higher levels of anxiety and depressive symptoms in ED patients. The severity of ED symptoms decreased to normal levels during the transition from lock-down to re-opening (Monteleone et al. 2021a, b). However, some participants reported relieving symptoms during the confinement period, especially for anorexia nervosa patients. The possible explanation is that patients continue to receive e-therapy during COVID-19 lockdown (Weissman et al. 2020), stable family relationships, and fewer social stressors might contribute to reducing symptoms of anorexia nervosa (Walsh and McNicholas 2020).

### Interpretation of the results

COVID-19 lockdown has placed restrictions on movement, which reduced access to usual supporting systems and healthcare services (Graell et al. 2020; Weissman et al. 2020), limited access to food, and negative emotions in people who suffer from eating disorder conditions.

### Social isolation and loneliness

Lockdown and the stay-at-home orders exacerbated social isolation and loneliness among this population (Smith and Lim 2020), especially for people who live alone. Most participants, except essential workers, were forced to work from home or were unemployed during the confinement period. People had limited interactions with anyone outside due to home isolation during the lockdown. Branley-Bell and Talbot (2020) highlighted that spending time with friends and relatives can be an important factor and motivation in the recovery process for ED patients. The alterations in the living situation during the confinement period increased social isolation and limited access to their usual support networks (Branley-Bell and Talbot 2020) and eventually led to worsened ED symptoms (Conceição et al. 2021).

### Limited access to healthcare

One of the major problems for ED patients during lockdown was reduced access to face-to-face treatment (Graell et al. 2020). Although some patients received online treatment (e.g., teletherapy and videoconferencing) during the confinement period, they stated that online support was an alternative but could not replace the traditional support mechanisms (Branley-Bell and Talbot 2020). During the COVID-19 pandemic peak, some psychiatric wards were downsized, closed, or converted into wards for coronavirus patients (Graell et al. 2020). ED patients experienced treatment suspension, cancellation of non-urgent treatment, and a reduction in hospital bed availability (Touyz et al. 2020; Branley-Bell and Talbot 2020).

### Dietary Changes & Food Restrictions

The lockdown measures placed restrictions on people’s movement and influenced the accessibility of food. Robertson et al. (2021) outlined that lockdown can impact people’s eating patterns and body image. The majority of participants have irregular eating patterns and experienced worsened ED symptoms during the COVID-19 outbreak (Chan and Chiu 2021; Alamrawy et al. 2021), which align with a previous study demonstrating the association between dietary changes and increasing eating disorders symptoms (Jacobi et al. 2004). Simone et al. (2021) highlights the common changes in eating behavior during the pandemic are mindless eating, increased or decreased appetite, and exposure to unhealthy food. The sudden change in eating habits can be one of the warning signs for ED symptomatology (Chan and Chiu 2021). COVID-19 lockdown measures can lead to a reduction in dietary intake, including dietary restrictions and loss of appetite as a coping strategy to stress levels (Simone et al. 2021; Polivy and Herman 2002). The finding is supported by Graell et al. (2020) and Rodgers et al. (2020) who noted that AN patients’ intolerance of uncertainty during the pandemic lockdown associated with the drive for thinness and body dissatisfaction can lead to weight-control behaviors to compensate for the fear of losing control in their daily routine (Brown et al. 2017; Frank et al. 2012; Tiggemann and Raven 1998) and deterioration in ED symptoms. Furthermore, financial difficulties during the COVID-19 lockdown might lead to food restrictions, unhealthy eating habits, depression, and anxiety symptoms, and result in increasing disordered eating symptoms (Simone et al. 2021).

### Psychosocial impact

Although lockdown restrictions were important to prevent the spread of COVID-19, it negatively impacted eating disorders and emotion control (Cecchetto et al. 2021). Monteleone et al. (2021a, b), Baenas et al. (2020), and Brooks et al. (2020) stated that COVID-19 and lockdown were characterized by fear of infection and uncertainty, boredom, social isolation, financial difficulties, and increasing mental health problems. Several studies mentioned anxiety and depressive symptoms were associated with increased odds of concurrent eating disorders (Simone et al. 2021; Chan and Chiu 2021; Meyer et al. 2011; Errisuriz et al. 2016). Negative emotions (e.g., sadness and fear) were important precursors for eating disorders patients (Naumann et al. 2014) and tend to have detrimental effects on restrictive eating and ED symptoms (Naumann et al. 2015). The risk of developing binge eating behaviors increased dramatically under negative emotions (Whiteside et al. 2005). Allison and Timmerman (2007) and Mason and Lewis (2017) concluded that context is vital in triggering eating disorder symptoms. Therefore, the combination of a health crisis (COVID-19 pandemic), social isolation (enforced lockdown), and negative emotions could lead to worsening ED symptoms and general psychopathology (anxiety and depression) in the population (Shanahan et al. 2020; Serafini et al. 2020).

### Contradicting findings

Fernández-Aranda et al. (2020a, b) observed a significant decrease in ED symptoms in AN patients after the confinement period. A possible explanation for this phenomenon is that patients were able to access support from e-therapy or follow the previous treatment plan. Patients who lived with families might have received increased social support and strengthened family relationships during the lockdown (Termorshuizen et al. 2020), resulting in alleviating ED symptoms. Especially for children and adolescents, as their parents might supervise their dietary intake (Graell et al. 2020; Walsh and McNicholas 2020).

### Limitations

The present study is limited by several factors. First, all identified studies were conducted in Europe (four in England, three in Italy, two in Spain, and one in Belgium and Portugal) and results may differ and not be generalizable to other settings. Second, the sample size was relatively small due to limited evidence on this topic. Lastly, lockdown measures have been different across countries, thus the impact on ED patients might differ.

## Conclusions and further directions

In general, coronavirus and lockdown restrictions were closely associated with worsening eating behaviors, depression, and anxiety symptoms among ED patients. The COVID-19 pandemic lockdown led to disruption of the living situation, increasing social isolation and loneliness, financial difficulties, and an unhealthy pattern of food consumption in the general population, which is positively associated with worsened ED symptoms. The majority of studies reported lockdown restrictions had a moderate to an extreme level of impact on life routines and many people experienced difficulties accessing healthcare services and mental healthcare, this triggering environment can create additional challenges for ED patients. However, some studies report positive outcomes during confinement and showed a recovery of anorexia nervosa symptoms. This might relate to accessing online treatment (Branley-Bell and Talbot 2020; Castellini et al. 2020; Weissman et al. 2020) and receiving support from their families (Graell et al. 2020; Walsh and McNicholas 2020).

Future systematic review studies need to examine the impact of lockdowns on other continents (e.g., Asia, Africa, North and South America, and Australia). In this systematic review, only one study included a reopening period for ED patients and concluded the disordered eating symptoms showed slow recovery from the impact of the COVID-19 lockdown (Monteleone et al. 2021a, b). Further research can extend the study period and explore the eating disorder symptoms pre and post COVID-19 lockdown, and the following re-opening period.

## Data Availability

All data supporting this systematic review are included in published articles.
